# Composite Fiber Spun Mat Synthesis and In Vitro Biocompatibility for Guide Tissue Engineering

**DOI:** 10.3390/molecules26247597

**Published:** 2021-12-15

**Authors:** Rodrigo Osorio-Arciniega, Manuel García-Hipólito, Octavio Alvarez-Fregoso, Marco Antonio Alvarez-Perez

**Affiliations:** 1 Laboratorio de Bioingeniería de Tejidos, DEPeI, Facultad de Odontología, Universidad Nacional Autónoma de México, Circuito Exterior s/n. Cd. Universitaria, Coyoacán 04510, Mexico; rosorioarci@gmail.com; 2 Instituto de Investigaciones en Materiales, Circuito Exterior s/n. Cd. Universitaria, Coyoacán 04510, Mexico; maga@unam.mx (M.G.-H.); oaf@unam.mx (O.A.-F.)

**Keywords:** composite scaffold, guide tissue engineering, polylactic acid (PLA) nanofibers, zirconium ceramic, biocompatibility

## Abstract

Composite scaffolds are commonly used strategies and materials employed to achieve similar analogs of bone tissue. This study aims to fabricate 10% wt polylactic acid (PLA) composite fiber scaffolds by the air-jet spinning technique (AJS) doped with 0.5 or 0.1 g of zirconium oxide nanoparticles (ZrO_2_) for guide bone tissue engineering. ZrO_2_ nanoparticles were obtained by the hydrothermal method and characterized by X-ray diffraction (XRD) and scanning electron microscopy (SEM). SEM and fourier-transform infrared spectroscopy (FTIR) analyzed the synthesized PLA/ZrO_2_ fiber scaffolds. The in vitro biocompatibility and bioactivity of the PLA/ZrO_2_ were studied using human fetal osteoblast cells. Our results showed that the hydrothermal technique allowed ZrO_2_ nanoparticles to be obtained. SEM analysis showed that PLA/ZrO_2_ composite has a fiber diameter of 395 nm, and the FITR spectra confirmed that the scaffolds’ chemical characteristics are not affected by the synthesized technique. In vitro studies demonstrated that PLA/ZrO_2_ scaffolds increased cell adhesion, cellular proliferation, and biomineralization of osteoblasts. In conclusion, the PLA/ZrO_2_ scaffolds are bioactive, improve osteoblasts behavior, and can be used in tissue bone engineering applications.

## 1. Introduction

Periodontal disease is a chronic inflammatory condition caused by a highly pathogenic biofilm. If left untreated, it may result in irreversible destruction of the supporting periodontal tissues, which consists of the hard and soft tissues surrounding and supporting the teeth. In severe cases, reduced periodontal support can lead to tooth loss, requiring tissue bone augmentation and regeneration, followed by surgical procedures to restore the reconstitution of the complex structure of the tissue [1].

In clinical applications, various attempts have been made to repair the bone/periodontal apparatus over the last four decades. These include root surface conditioning, graft materials, non-resorbable membranes of polytetrafluoroethylene (e-PTFE), and bioceramic scaffolds [2]. However, the regeneration of defective or damaged bone/periodontal tissue has been challenging in reconstructive surgery. Furthermore, it is still considered a common cause of permanent functional loss and post-traumatic morbidity [3].

Hence, guiding the regeneration processes of the bone/periodontal apparatus’s complex architecture still represents one of the most significant challenges in modern dentistry. From an anatomical and physiological perspective, the functional integration of composite scaffolds and/or polymeric matrices doped with nanoparticles that synchronously guide the tissue’s regeneration is still demanding [4].

In recent years, scaffolds that combine a biodegradable polymers matrix, such as PCL, PLGA, or PLA doped with bioceramics (i.e., hydroxyapatite, TiO_2_, magnesium-calcium silicate, β-Tricalcium phosphate, mineral trioxide aggregate, calcium phosphate) have received considerable attention as promising biomaterials with potential for next-generation bone tissue engineering scaffolds, since they provide peculiar bioactive signals to improve osteoconductivity and to enhance mesenchymal stem cells adhesion, migration, and differentiation [5,6,7,8,9,10]. In addition, among the different calcium ceramics used in the application in bone and periodontal surgery, there is also the use of zirconium oxide (ZrO_2_) due to its excellent physicochemical properties, its high chemical and dimensional stability, its low ionic and thermal conductivity, its excellent mechanical resistance, fracture toughness, its low cytotoxicity, and its biocompatibility response [11,12]. Moreover, the use of the thermoplastic aliphatic polyester as polylactic acid (PLA) in bone tissue engineering is because it has numerous interesting properties, including excellent mechanical properties, thermal stability, processability, and low environmental impact. However, its most important characteristic is that it is Food and Drug Administration (FDA) approved for the low toxicity and degradation products (H_2_O and CO_2_) that do not interfere with tissue healing. Indeed, the hydrolyzes of the constituent of PLA could be incorporated into the tricarboxylic acid cycle and excreted, making it a natural choice for biomedical applications [13,14].

Recently, the synthesis of polymeric nano- or microfibers with bioceramics via electrospinning has been widely applied in bone tissue engineering; due to its large surface/volume ratio, the process allows modification of their physical or chemical properties and enables precise control over the shapes and structures of the fibers, which is often reported in the literature [8,15,16,17,18,19]. Likewise, another versatile, low-cost, safe (does not employ high voltage), scalable, and versatile method that produces micro/nanoscale fibers from different synthetic and natural polymers is the air-jet spinning (AJS) method. This method utilizes a specialized spinning system nozzle, such as a commercial airbrush, a surface for collecting polymer fibers, and compressed gas through which the polymer solution and a pressurized gas are simultaneously ejected to form the fiber morphology, allowing the design of sheet-like platforms that could be applied in bone and tissue engineering [20,21,22,23]. Furthermore, poly(lactic acid) fiber scaffolds were successfully fabricated, and our in vitro biological response of mesenchymal stem cells showed that the polymer concentration and fiber size influenced the biocompatibility response. Furthermore, our results indicated that nanofibrous topography possesses the potential to enhance cell adhesion and proliferation and improve the cues to guide the fiber orientation by the cells; additionally, our in vivo studies showed that PLA fiber spun scaffolds are not cytotoxic in a Wistar rat model [24,25,26]. Moreover, our previous results, where we reported the synthesis of polymer composites composed of PLA/ZrO_2_ to attempt optimization of the fabrication by AJS, showed that composites have a fibrous morphology with a random distribution, with a diameter of fibers and mechanical properties depending on polymer and zirconia concentrations, suggesting that the PLA/ZrO_2_ composite may be used as a biomaterial [27]. Thus, we propose the preparation of a composite by using a polymer solution of 10% wt of PLA with 0.1 and 0.5 g of ZrO_2_ nanoparticles by the AJS method, with the final goal of finding a biomaterial for bone/periodontal tissue engineering, with the presence of bioceramic as an environmental cues signal needed to guide osteoblast cell responses.

## 2. Results

### 2.1. Characterization of ZrO_2_ Nanoceramic

**Figure 1:**
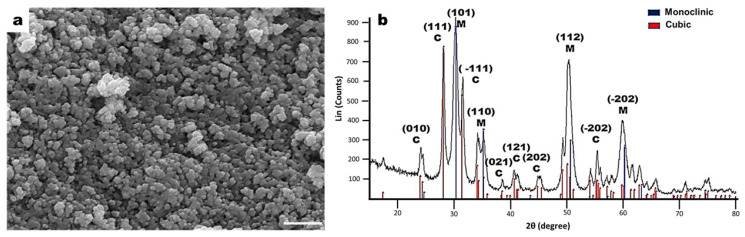
Characterization of zirconium oxide (ZrO_2_) nanoceramic. (**a**) Scanning electron microscopy images of zirconium oxide nanoparticles. ZrO_2_ nanoparticles with homogeneous morphology and semispherical surface; the nanoparticles tend to agglomerate; scale bar = 200 nm. (**b**) X-ray diffraction patterns of ZrO_2_ nanoparticles obtained with incident angles at 30.5°, 31.7°, 35.2°, 50.2°, 60.1°, and 63.2°.

A hydrothermal technique was used to synthesize zirconium oxide nanoparticles. This allows a nanoceramic with homogeneous semispherical morphology, with dimensions of around 25 to 80 nm, to be obtained; this could be due to the presence of agglomerates constituted by several small polycrystallites in the range of 10–20 nm in the dimension (Figure 1a). Moreover, the X-ray diffraction (XRD) pattern showed a considerable broadening of the peaks due to the nanostructure of the crystalline grains (Figure 1b). The XRD of the ZrO_2_ showed a crystalline behavior with two phases. Based on the XRD spectrum of the JCPDS card No. 03-065-0461, the monoclinic phase showed the peaks (1 0 1) at 30.26°, (1 1 0) at 35.31°, (1 1 2) at 50.28° and (−2 0 2) at 62.93°. A cubic phase was also identified, with peaks (0 1 0) at 24°, (1 1 1) at 28°, (−1 1 1) at 31.5°, (0 2 1) at 38.5°, (1 2 1) at 41°, (2 0 2) at 45.25°, (−2 0 2) at 55°, and (3 1 1) at 60.28°, according to the JCPDS card No. 03-065-0461. Using the Debye-Scherrer formula for the line broadening fitting curve XRD program, the particle size was evaluated. The average particle diameter for the monoclinic phase was around 25–35 nm, considering that the grains were spheres. For the cubic phase, the average size was estimated at around 6–14 nm.

### 2.2. Characterization of PLA/ZrO_2_ Nanocomposite Fiber Scaffold

**Figure 2:**
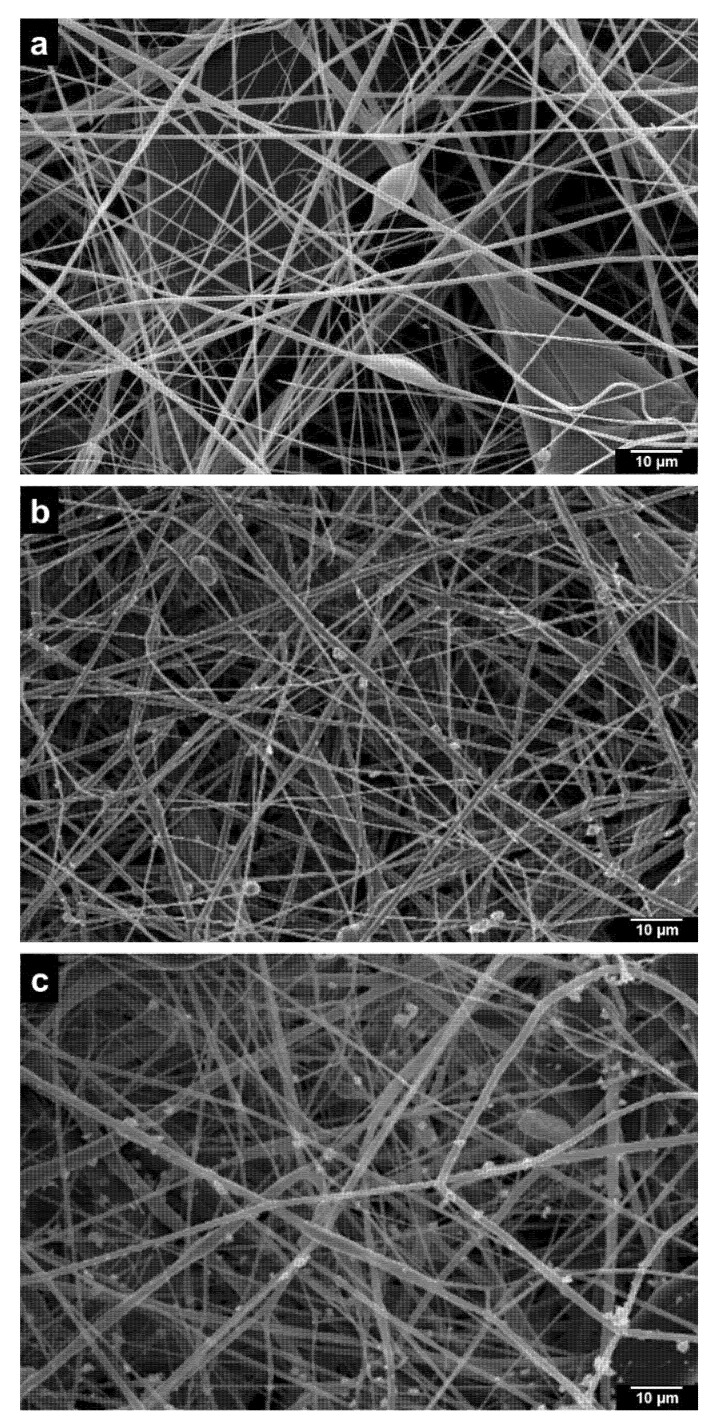
Scanning electron microscopy assessment of the microstructure of the polylactic acid (PLA) scaffolds loaded with different concentrations of zirconium oxide nanoparticles (ZrO_2_). (**a**) PLA scaffolds synthesized with the air-jet spinning technique. (**b**) PLA scaffold loaded with 0.1 g of ZrO_2_. (**c**) PLA scaffold loaded with 0.5 g of ZrO_2_. The PLA/ZrO_2_ scaffolds show dispersed and unsaturated ZrO_2_ nanoparticles along the PLA fibers, scale bar = 10 µm.

Figure 2 shows the analysis of the fiber membrane scaffolds of 10% wt of PLA and PLA/ZrO_2_ nanocomposite fiber scaffold by scanning electron microscopy (SEM). The PLA fiber scaffold is composed of smooth and uniform fibers with minimal bead formation and a diameter of around 400 nm with random orientation (Figure 2). The morphological analysis demonstrated that PLA/ZrO_2_ scaffolds have a rough surface due to the zirconium nanoceramic. The particles are observed through the fiber and sometimes as agglomerates around the interconnected strands with random orientation (Figure 2b,c). The analysis of the diameter sizes of the PLA/ZrO_2_ nanocomposite fiber scaffold showed that incorporating the nanoceramic (0.1 and 0.5 g) increased the fiber diameter. Moreover, the fibers were in the range of 100 to 800 nm, with an average diameter of 395 nm.

**Figure 3:**
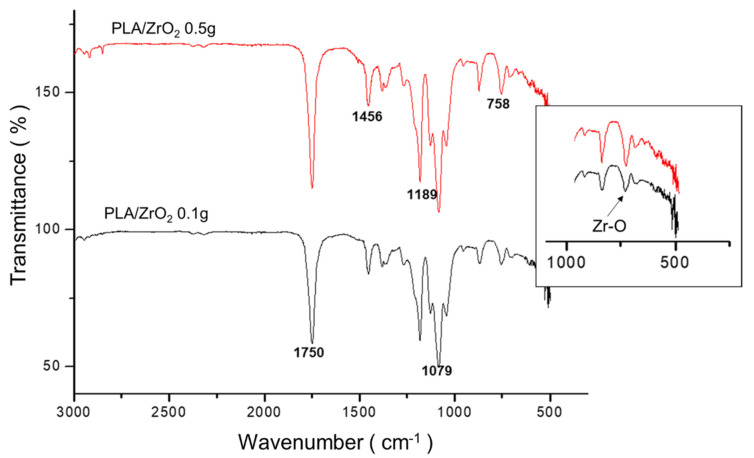
FTIR-IR spectrum of PLA/ZrO_2_ fiber membrane scaffolds. The absorption bands typical characteristic of PLA: around 1750 cm*^−^*^1^, the (C=O) ester carbonyl group; at 1445 and 1380 cm*^−^*^1^, the absorbance bands of C-H bending vibration of CH_3_; at 1350 cm*^−^*^1^, the bending vibration of carbonyl CH; at 960 to 830 cm*^−^*^1^, corresponding to the backbone stretching and CH_3_ rocking; at the region of 3200 to 2800 cm*^−^*^1^, to the symmetric and asymmetric stretch of CH; and at 1260 cm*^−^*^1^ and 1100 cm*^−^*^1^, corresponding to the lactide C-O stretch, with 1090*–*1189 cm*^−^*^1^ (C-O=C) and 758 cm*^−^*^1^ attributed to (Zr–O). The insert image of the absorption band of Zr-O at 758 cm*^−^*^1^ was magnified for show the differences between the PLA/ZrO_2_ with 0.1 g and 0.5 g composite scaffold.

The chemical structures of PLA fiber membranes synthesized with different concentrations of the ZrO_2_ nanoceramic (0.1 and 0.5 g) were obtained using fourier-transform infrared spectroscopy (FTIR) spectroscopy. The results were compared to identify structural changes by incorporating the nanoceramic onto the PLA polymer matrix (Figure 3). The infrared absorbance spectra showed the typical characteristic of PLA; i.e., the absorption bands around 1750 cm^−1^ corresponding to the (C=O) ester carbonyl group; at 1445 and 1380 cm^−1^, corresponding to the absorbance bands of the C-H bending vibration of CH_3_; at 1350 cm^−1^ corresponding to the bending vibration of carbonyl CH; at 960 to 830 cm^−1^, corresponding to the backbone stretching and CH_3_ rocking; at the region of 3200 to 2800 cm^−1^, corresponding to the symmetric and asymmetric stretch of CH; and at 1260 cm^−1^ and 1100 cm^−1^, corresponding to the lactide C-O stretch. However, the typical spectral of PLA was accompanied by the absorption band peaks of the ZrO_2_ nanoceramic; especially, a band at 758 cm^−1^ without structural change on the PLA fiber after doping with the nanoceramic.

### 2.3. Biocompatibility Assay

**Figure 4:**
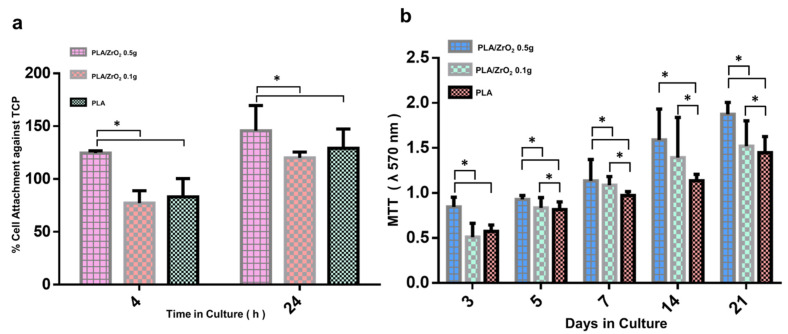
The hFOB biological response, after culture onto the PLA/ZrO_2_ scaffolds. (**a**) The cellular adhesion response of hFOB at 4 and 24 h, evaluated with violet crystal. The cell adhesion onto the PLA/ZrO_2_ nanocomposite fiber scaffold surface is presented as the percentage of attached cells in relation to control tissue culture plates. (**b**) Metabolic activity was evaluated with MTT assay at 3, 5, 7, 14, and 21 days to confirm that the PLA/ZrO_2_ nanocomposite fiber scaffold is not toxic to the cells. The viability of the hFOB cell increased time-dependently, with the best results observed for the PLA/0.5 g of ZrO_2_ after 21 days of culture. Asterisk (*) mean that scaffolds showed a statistical significance (*p* < 0.05).

The biocompatibility of the PLA/ZrO_2_ nanocomposite fiber scaffold was analyzed in in vitro cell culture to investigate the cell adhesion and cell viability of hFOB 1.19 cells. The cellular adhesion response of hFOB 1.19 cells at 4 and 24 h over the surface of the PLA/ZrO_2_ nanocomposite fiber scaffold are presented as the percentage of attached cells in relation to control tissue culture plates (Figure 4a).

The cell adhesion of hFOB 1.19 cells was favorable. It exceeded 100% at 4 h and ≥150% at 24 h of attachment onto the PLA/ZrO_2_ doped with 0.5 g of the nanoceramic with statistical differences compared to the PLA/ZrO_2_ doped with 0.1 g of the nanoceramic and the PLA fiber spun mat at *p* < 0.05. However, there were no statistical differences between the adhesion of hFOB 1.19 cells onto the PLA/ZrO_2_ doped with 0.1 g of the nanoceramic and the PLA fiber spun mat. The cell attachment for the former conditions was around 80% at 4 h and ≥120% at 24 h.

Concerning the cell viability, we performed the MTT assay to confirm that the PLA/ZrO_2_ nanocomposite fiber scaffold is not toxic to the cells (Figure 4b). The results are presented as the optical absorbance at 570 nm. The histogram in Figure 4b suggests that, in all scaffolds, high levels of MTT oxidation are present. However, the higher conversion rate of MTT was found in PLA/ZrO_2_ doped with 0.5 g of the nanoceramic from day 3, and with a constant increment until 21 days of cell culture. Furthermore, the MTT conversion rate of the PLA/0.5 g ZrO_2_ scaffold was followed by PLA/ZrO_2_ doped with 0.1 g of the nanoceramic and by the PLA fiber scaffold. Furthermore, statistical differences were found between the viability of hFOB cell culture onto the PLA/ZrO_2_ nanocomposite fiber scaffold and hFOB cell culture in the PLA fiber scaffold at *p* < 0.05.

### 2.4. Cell-Material Interaction

**Figure 5:**
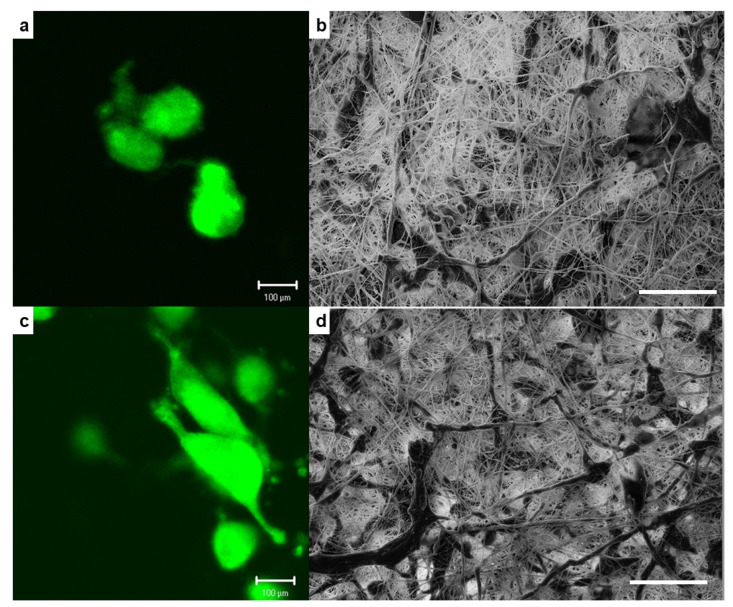
Representative fluorescence images of the cellular morphology and SEM micrographs of the spreading pattern interaction of hFOB cells cultured onto PLA fiber scaffold (**a**,**b**) and PLA/0.5 g ZrO_2_ scaffolds (**c**,**d**) after 24 h of culture. Scale bar on SEM images = 100 μm.

Figure 5 showed the cell morphology and the cell spreading pattern interaction between hFOB cells onto the PLA fiber scaffold and the PLA/ZrO_2_ composite fiber scaffold doped with 0.5 g of the nanoceramic. The fluorescence images of the morphology showed that human osteoblasts cultured onto PLA/ZrO_2_ had a well-attached cell with elongated morphology and filopodia extensions (Figure 5c,d), in comparison with the less spreading and less elongated morphology shown by osteoblasts cultured onto the control PLA fiber scaffold (Figure 5a,b). Additionally, SEM micrographs showed that cells onto PLA presented a rounded cytoplasm, with few spreading cells that exhibited a flat shape (Figure 5b), compared with preferential spread on the entire surface of the PLA/ZrO_2_ composite fiber scaffold, wherein some points present an elongated morphology in direct contact with the nanofiber morphologies of the scaffolds due to the presence of cues exerted by zirconia nanoparticles (Figure 5d).

### 2.5. Biomineralization Assay

**Figure 6:**
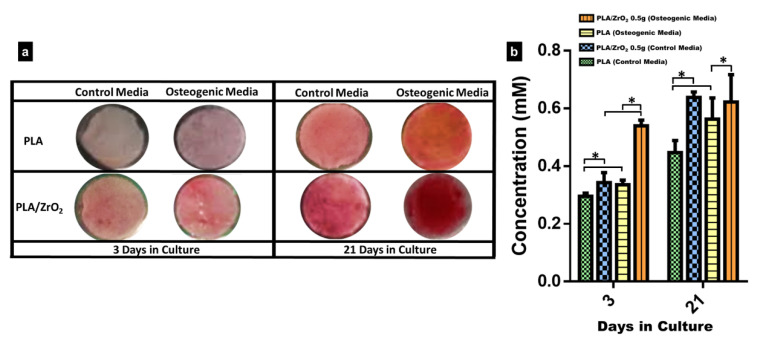
Alizarin red staining. The hFOB cells were cultured onto the PLA and PLA/ZrO_2_ with the presence or absence of an osteogenic medium. (**a**) The calcified extracellular matrix deposits produced by hFOB cells onto the PLA/0.5 g ZrO_2_ composite scaffolds are red. The area of the calcified deposits onto the scaffolds was time-dependent and increased with the culture time. However, the calcium deposit onto the PLA/0.5 g ZrO_2_ scaffolds in the osteogenic medium, compared with the standard medium, was more visible, and the concentration of the alizarin was higher than in the PLA scaffolds. (**b**) The calcium (mM) concentration in PLA/0.5 g ZrO_2_ scaffolds after 3 and 21 days of culture in osteogenic medium and standard medium. Asterisk (*) mean that the scaffolds showed a statistical significance (*p* < 0.05).

For the analysis of the biomineralization assay, we selected only the PLA/ZrO_2_ of 0.5 g scaffold because it demonstrated the best results for cell adhesion and cell viability. The bioactivity of hFOB cells onto the composite was analyzed by Alizarin Red S staining (ARS) after 3 and 21 days (Figure 6). The images of light microscopy showed that after three days of culture, on control media, there was almost a very weak staining onto the PLA scaffold and the PLA/ZrO_2_ composite fiber scaffold, in comparison with a very light signal of the red staining in both the PLA spun mat and the PLA/ZrO_2_ composite fiber scaffold cultured on osteogenic media. However, after 21 days of culture, on control media, redder staining onto the PLA scaffold and the PLA/ZrO_2_ composite fiber scaffold could be seen, in comparison with a darker red precipitate throughout the surface on both the PLA fiber scaffold and the PLA/ZrO_2_ composite fiber scaffold, which indicates calcium deposits (Figure 6a). Likewise, the semi-quantitative analysis of the red alizarin staining reveals that the PLA/ZrO_2_ composite scaffold presents a higher concentration related to the calcium precipitates at 3 and 21 days of culture than the PLA fibers in both culture media (Figure 6b).

**Figure 7:**
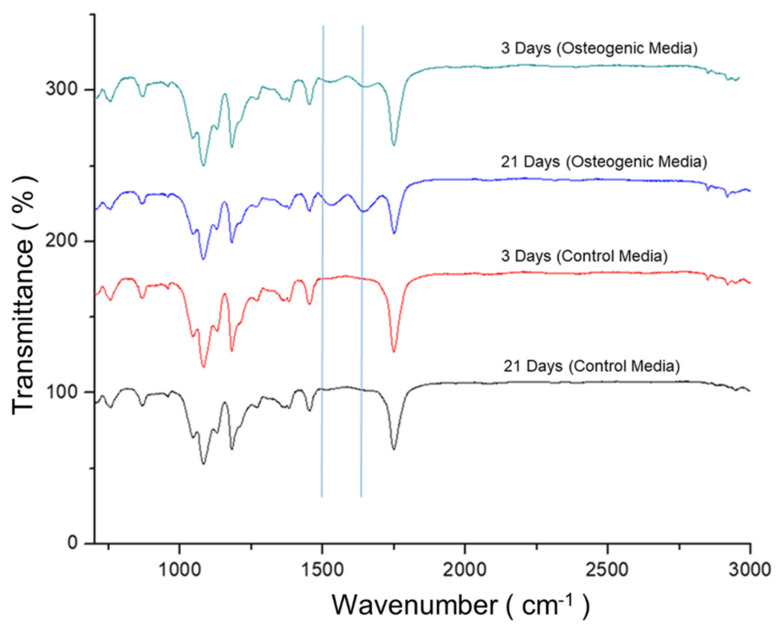
FTIR-IR spectrum of PLA/ZrO_2_ scaffolds after 3 and 21 days of culture with hFOB. The PLA/ZrO_2_ scaffolds seeded with hFOB cells were cultured with and without the presence of the osteogenic medium. The PLA/0.5 g ZrO_2_ scaffold spectrum after 3 and 21 days of culture with osteogenic medium showed new peaks that are characteristic of the amide I and amide II groups at 1650 cm^−1^ and 1533 cm^−1^; the bands that correspond to the PLA are also present.

Moreover, the PLA scaffold and PLA/ZrO_2_ composite fiber scaffold were analyzed by FTIR after 3 and 21 days of culture in osteoinductive culture media and compared to the control culture. From the spectra, the peaks that are characteristic of the amide I and amide II groups could be observed at 1650 cm^−1^ and 1533 cm^−1,^ corresponding to the extracellular collagen matrix (Figure 7).

## 3. Discussion

Tissue engineering (TE) is a multidisciplinary area of research aimed at repairing, replacing, or regenerating tissues or organs to restore impaired function due to defects and aging. Nanotechnology for TE application focuses on imitating the size and role of the extracellular matrix (ECM) with a biomimetic characteristic for improving the migration, proliferation, and differentiation of cells [28,29]. This ECM biomimetic related to bone tissue could be obtained by synthesizing a scaffold with a combination of different biomaterials as polymers and bioceramic, via several manufacturing methods that allow the combining of its properties for translation to the new composite phase, to improve its functionality [5,6,7,8,9,10,30]. Thus, in this study, we used hydrothermal synthesis and air-jet spinning (AJS) technology to engineer a multifunctional composite scaffold as a platform for bone tissue engineering applications.

First, we used hydrothermal synthesis, defined as a homogeneous reaction in the presence of aqueous solvents under temperature and pressure conditions to dissolve and recrystallize insoluble materials under normal conditions, to obtain the fiber’s ceramic phase composite scaffold. Our XRD and SEM images show that hydrothermal synthesis allows a homogeneous thermally stable crystalline nanoceramic of ZrO_2_ to be obtained. Furthermore, the nanoceramic showed agglomerates of crystallites, of nearly uniform size, between 10–80 nm on average for the particle diameter, with nanometric dimensions considering that the grains are spheres, and the results showed that nanocrystals coincide with the monoclinic and cubic structural phases specific to ZrO_2_; the results are consistent with previous studies reported [31,32,33].

For bone application, nanocomposite fiber biomaterials are a relatively new class of materials that combine a homogeneous size of the crystalline structure with biopolymeric matrix structure, that enables the improvement of the fibrillar design nanocomposite [34,35]. In this regard, we explore the use of AJS to design a polymeric fibrillar matrix of 10% wt of PLA, doped with two different concentrations (0.1 and 0.5 g) of the nanoceramic of ZrO_2_. As demonstrated by our results, the SEM images of the PLA/ZrO_2_ nanocomposite had a random orientation of the fiber morphology with a submicron/nanometer range between 100 nm up to 1 µm in diameter, with an average size of 395 nm; by FTIR, it was possible to detect differences between the intensity peak of 0.5 and 0.1 g of ZrO_2_ that could be related to the mass fraction of the nanoceramic for 0.1 g (~0.0099 g) and for 0.5 g (~0.047 g) in the composite. This difference in the peak intensity of samples showed that, with increased concentrations of nanoceramic, the corresponding interaction of the Zr-O bonds with the characteristic bands of the functional groups of PLA (carbonyl group, esters CH and CH_3_) at 758 cm^−1^ could be detected; furthermore, this different concentration improves the anisotropic properties of the scaffold that could be related to the adhesion between the interface of the fiber matrix and the nanoceramic particles, in concordance with previous studies reported [25,26,27,36,37].

In bone tissue engineering, the goal of a nanocomposite is intended to provide a suitable surface for supporting the tissue regeneration process, and therefore requires osteoconduction or osteoinduction properties [35]. Our study found that the doped polymer matrix with the nanoceramic of ZrO_2_ could be considered adequate to the bone because the result showed appropriate surface property cues and physicochemical stability that have been defined as important parameters for influencing cell cytocompatibility and osteogenesis [37,38,39,40]. In our study, we analyzed the biocompatibility of the PLA/ZrO_2_ composite fiber scaffold by cell adhesion and viability response of human osteoblasts cells hFOB 1.19. In the adhesion assay and viability evaluation, we found that the composite scaffold of PLA doped with 0.5 g of the nanoceramic had higher cell attachment and proliferation of cells. This cellular behavior could be influenced by the optimal surface topography, the physicochemical properties of the material, the nanometric surface features, and the critical size of the doped nanophase that plays a role in mediating the cell behavior response [14,41].

Moreover, the physical, chemical, and surface topography property cues are critical parameters that induce cellular recognition signals. Our results showed that hFOB cells attached well to the fiber composite, covering long area extensions with very spreading, elongated, and lamellae morphology. Thus, the morphology of the topographic cues (spherical nanostructure) of the nanocomposite of PLA related to the presence of ZrO_2_ demonstrated an excellent surface that stimulated cell attachment and proliferation and supported the extracellular matrix deposit by the osteoblast cells, as shown by alizarin red staining. This is an important effect of the nanoscale because AJS allows the synthesis of a more suitable scaffold with the physical integration of nanoceramic, conferring a homogenous distribution for cell binding. Furthermore, these could be improved by the adsorption of biomolecules by the media that can activate signaling pathways that change the cell behavior, recreating a microenvironment similar to the native tissue, indicating the recognition or sense of the surface by the osteoblast cells, favorably inducing a good cell-material interaction and differentiation [13].

In our study, we evaluated the potential of the 0.5 g of PLA/ZrO_2_ fiber nanocomposite in promoting the mineral deposit of the ECM of the hFOB by a differentiation assay. Our analysis by alizarin red staining (ARS) and FTIR showed that osteoblasts could deposit a mineral-like tissue over the nanocomposite surface after 21 days of culture. This indicates the importance of the chemical components at the nanoscale, and the submicron-sized topographical cues that could modulate the cell osteoblasts behavior. Moreover, the chemical component related to the insertion of the ZrO_2_ nanoceramic onto the fiber polymer matrix may confer a possible cue role on the bioactivity related to the osteogenesis potential that influences the proliferation and differentiation. The mineral deposit evaluated by ARS and FTIR results indicate that topographical properties of the nanocomposite were related to the size and morphology of the fiber, the porosity, but also to the osteogenic stimuli (inductive factors) presence on the cell culture media, together performing a specific role supporting osteoblast cell differentiation. Thus, our results are in agreement with different studies that reported that zirconia nanofibers or ZrO_2_ composites may have pro-osteogenic properties, have good mechanical biocompatibility, and improve the metabolism of bone precursor cells. Furthermore, they could also be considered as a self-regulation material that possesses an excellent osteoinductive capacity that could modify the deposition of ECM by bone cells during differentiation or in response to the surface material, because the osteoblast cell functions could be substrate-sensitive, either with respect to stiffness, roughness, or to the size of the composition of the material [16,17,42,43,44,45,46].

## 4. Materials and Methods

### 4.1. Synthesis of Nanoceramic of Zirconium Oxide

Zirconium oxide nanoceramic was synthesized via the hydrothermal technique. The volume ratio of zirconium tetrachloride (ZrCl_4_) and sodium hydroxide (NaOH) was 3:1. The obtained solution was mixed with pH 14 and let to settle for 20 h, until a substitution reaction happened between ZrCl_4_ and NaOH, obtaining sodium chloride (NaCl) and zirconium oxide (ZrO_2_) as products. After that, the reaction product was removed, washed five times with deionized water, then the clean ZrO_2_ was loaded into a Teflon vessel for autoclaving. Afterward, the autoclave was placed in a furnace (Barnstead model 1500), heated, and maintained at 200 °C for 2 h, then cooled to room temperature. The powder obtained was crushed on an agate mortar. The characterization of the ceramic was made by X-ray diffraction analysis and scanning electron microscopy (SEM).

The crystal size was calculated by the equation of Scherrer, where d is the mean size of the particle, *K* is the Scherrer constant, *λ* is the wavelength, *β* is the peak at half the maximum intensity (FWHM), and *θ* is the Bragg angle (in degrees). The following equation is applied only for crystals with sizes in the range of 1–100 nm.

$$d=\frac{K\lambda }{\beta \;cos\theta }$$

### 4.2. Polymer Fiber Composite Fabrication of PLA/ZrO_2_

Fibrous composite spun scaffolds were fabricated via the AJS process from PLA polymeric solutions of 10% wt. First, polymeric solutions of 10% wt of PLA were prepared: PLA pellets (C_3_H_6_O_3_; MW 192,000 from Nature Works, Minnetonka, MN, USA) were dissolved in chloroform (CHCl_3_ from J. T. Baker) and stirred for 20 h. After that, acetone was added, and the solution was stirred for 30 min to obtain a homogeneous solution. The volume ratio of chloroform/acetone was 3:1. Then, the polymeric solution was prepared with two different amounts of nanoceramic of ZrO_2_ (0.1 and 0.5 g) for composite scaffolds. During their preparation, solutions were incubated in an ultrasonic bath for 1 h to guarantee the homogenization of nanoparticles onto the polymeric solution. In all cases, the polymeric solution was placed in a commercially available airbrush ADIR model 699 with a 0.3 mm nozzle diameter and with a gravitational feed of the solution to synthesize the fiber membrane scaffolds. The airbrush was connected to a pressurized argon tank (CAS number 7740-37, concentration > 99%, PRAXAIR Mexico, Nuevo Leon, Mexico). For deposition of the fibers, a pressure of 30 psi with 11 cm of distance from the nozzle to the target was held constant. The fiber scaffold characterization was analyzed by a Fourier transform infrared (FTIR) spectrophotometer, employing an IRAffinity-1S (Shimadzu, Kyoto, Japan) within the 400–4000 cm^−1^ range. The morphology and structure of the fibers were observed with a field emission scanning electron microscope (FE-SEM, JSM-7800F, JEOL). Fiber diameter was measured from SEM micrographs employing image analysis software (ImageJ, National Institutes of Health, Gaithersburg, MD, USA).

### 4.3. Cell Culture

The human fetal osteoblast cell line (hFOB 1.19, ATCC CRL-11372) was used to evaluate the cell biocompatibility response of the PLA fibers spun mat, and the PLA/ZrO_2_ spun composite scaffolds. Briefly, hFOB 1.19 cells were cultured in 75 cm^2^ culture flasks, in Ham’s F12/Dulbecco’s modified Eagle’s Media (1:1, Ham’s F12-DMEM, Sigma-Aldrich, St. Louis, MO, USA), supplemented with 10% fetal bovine serum (FBS, Biosciences, Collierville, TN, USA), 2.5 mM L-glutamine, and an antibiotic solution (streptomycin 100 μg/mL and penicillin 100 U/mL, Sigma-Aldrich, St. Louis, MO, USA). The cells were incubated in a 100% humidified environment at 37 °C in 95% air and 5% CO_2_. The hFOB 1.19 cells were used between 2–6 passages of culture for all the experimental procedures.

### 4.4. Cell Adhesion

As the first attempt to investigate how hFOB cells would interact with the PLA/ZrO_2_ nanocomposite fiber material, adhesion assays were performed. For this purpose, hFOB 1.19 cells were seeded at 1 × 10^4^ cells/mL onto the composite scaffold in triplicate and were allowed to adhere under standard cell culture conditions for 4 and 24 h. After each time, nanocomposite fiber scaffolds were rinsed three times using phosphate-buffered saline (PBS) to remove non-adherent cells. Next, the adherent cells were fixed with 3.5% paraformaldehyde, and the evaluation of cell attachment was performed according to the crystal violet assay. Briefly, cells were incubated with 0.1% crystal violet solution for 15 min and then washed carefully with deionized water until clear water was obtained. Then, the dye was extracted with 0.1% of sodium dodecyl sulfate (SDS), and the optical absorption was quantified by spectrophotometry at 550 nm with an ELISA plate reader (ChroMate, Awareness Technology). Cells attached in triplicate onto a conventional tissue culture plate (TCP) were considered as 100% cell attachment of hFBO 1.19 cells, and cells seeded in triplicate onto PLA fiber membrane scaffolds were used as the control.

### 4.5. Viability Cells (MTT Assay)

The cell viability response of hFOB 1.19 cultured onto PLA/ZrO_2_ composite fiber membrane scaffolds in triplicate was analyzed with the MTT assay. The assay is based on the ability of mitochondrial dehydrogenases to oxidize thiazolyl blue (MTT), a tetrazolium salt (3-[4,5-dimethylthiazolyl-2-y]-2,5-diphenyltetrazolium bromide), into an insoluble blue formazan product. Cells were seeded at 1 × 10^4^ cell/mL density in triplicate and incubated for 3, 5, 7, 14, and 21 days of culture. After each time, PLA/ZrO_2_ nanocomposite fiber scaffolds were washed with PBS and incubated with fresh cultured medium containing 0.5 mg/mL of MTT for 4 h at 37 °C in the dark. Then, the medium was removed, and 500 µL of dimethyl sulfoxide (DMSO) was added to each well. After 60 min of slow shaking, the absorbance was read at 570 nm. Cells seeded in triplicate onto PLA fiber membrane scaffold were used as the control. During the experimental time, the culture medium was changed every two days.

### 4.6. Cell-Material Interaction

We examine the cell morphology, the spreading patterns, and cell-material interaction of hFOB 1.19 cells cultured onto the PLA fiber scaffold and PLA/ZrO_2_ of 0.5 g nanocomposite fiber scaffold, because it demonstrated the best results for adhesion and viability. Briefly, hFOB 1.19 cells were cultured at a density of 1 × 10^4^ cells/mL onto the scaffolds for 24 h in triplicate. Then, the samples were analyzed using SEM and fluorescence microscopy (AMSCOPE). For SEM analysis, after 24 h of incubation, scaffolds were washed three times with PBS, fixed with 4% formaldehyde, then dehydrated with a graded series of ethanol (25–100%) and air-dried. Next, the samples were sputter-coated with a thin layer of gold and examined by SEM. For fluorescence observation, before seeding the hFOB 1.19 cells onto the scaffolds, cells were incubated with CellTracker™ Green (CMFDA, 5-chloromethylfluorescein diacetate) in phenol red-free medium at 37 °C for 30 min. Subsequently, the cells were washed with PBS and incubated for 1 h in a complete medium. Then, hFOB 1.19 cells were trypsinized and counted to the desired cell concentration (1 × 10^4^ cells/mL), incubated for 24 h onto the scaffolds, and examined under the microscope.

### 4.7. Biomineralization Assay

As previously mentioned, the PLA/ZrO_2_ of 0.5 g nanocomposite fiber membrane scaffold demonstrated the best results in the biocompatibility assays. Therefore, it was used for biomineralization assays. Briefly, hFOB 1.19 cells were seeded onto the PLA fiber scaffold and PLA/ZrO_2_ of 0.5 g scaffold at a density of 1 × 10^4^ cell/mL and left to adhere overnight. Then, a group of scaffolds (PLA and PLA/ZrO_2_) were incubated with mineralizing media (complete medium, 50 µg/mL of ascorbic acid, 10 mM glycerol-2-phosphate, and 10^−7^ M of dexamethasone) and, together with the control group (PLA and PLA/ZrO_2_ scaffolds), were maintained in standard media without any osteogenic factors for 3 and 21 days of culture. After a time, PLA and PLA/ZrO_2_ composite fiber membrane scaffolds were washed three times with PBS. Then, cells were fixed with 4% formaldehyde for 1 h, washed five times carefully with distilled water, and then stained with a saturated solution (40 mM) of Alizarin Red S pH 4.2 for 30 min at room temperature. After several washes with distilled water to remove the dye excess, scaffolds were examined under an optical microscope.

For the quantitative analysis of ARS staining, the fiber membrane scaffolds were transferred to a 1.5 mL microcentrifuge tube, and ARS was extracted with 10% acetic acid, incubated for 30 min, and then heated at 85 °C for 10 min. Samples were then cooled for 5 min on ice and centrifuged at 20,000 rpm for 15 min; the reaction was stopped with 10% ammonium hydroxide. Finally, the dye solution sample was read at 405 nm in the spectrophotometer. The concentration of ARS was determined by correlating the absorbance of the experimental samples with a standard known curve of ARS dye concentrations. The experiments were repeated twice, and three fiber scaffolds were used in each experiment. Moreover, the mineral-like tissue deposited onto the PLA and PLA/ZrO_2_ composite fiber membrane scaffold at 3 and 21 days of culture was analyzed by FTIR.

### 4.8. Statistical Analysis

Statistical analysis was performed using a two-way ANOVA (GraphPad Prism 7 software) followed by Tukey’s post-hoc test to evaluate the differences among groups. All the results are presented as means ± standard deviation. A *p*-value of less than 0.05 was considered statistically significant.

## 5. Conclusions

In summary, our results indicate that thermally stable ZrO_2_ nanoceramic was successfully synthesized via the hydrothermal method with an average size of 10 to 35 nm. The air-jet spinning technique allowed the design of a nanocomposite scaffold of PLA/ZrO_2_ composed of fiber morphology, with an average diameter of 395 nm, with random distribution. The biocompatibility of osteoblasts analyzed in the function of cellular adhesion and cellular viability onto the PLA/ZrO_2_ fiber nanocomposite did not show any cytotoxic effects. Moreover, 0.5 g of the PLA/ZrO_2_ nanocomposite influenced a good cell functionality, providing a microenvironment correlated to the nanometric and interconnectivity of the fiber scaffold that increased the cell bioactivity over the topography, shown by the mineral-like deposit. However, further studies may be focused on the structural properties of the 0.5 g PLA/ZrO_2_ fiber nanocomposite in critical-size bone models as a future potential to be applied in bone tissue engineering.

## Data Availability

All data generated for this study are included in the manuscript.
